# Circadian entrainment in Arabidopsis

**DOI:** 10.1093/plphys/kiac204

**Published:** 2022-05-04

**Authors:** Shouming Wang, Gareth Steed, Alex A R Webb

**Affiliations:** Department of Plant Sciences, University of Cambridge, Downing Street, Cambridge CB2 3EA, UK; School of Life Science and Technology, Hubei Engineering University, Xiaogan 432000, China; Department of Plant Sciences, University of Cambridge, Downing Street, Cambridge CB2 3EA, UK; Department of Plant Sciences, University of Cambridge, Downing Street, Cambridge CB2 3EA, UK

## Abstract

Circadian clocks coordinate physiology and development as an adaption to the oscillating day/night cycle caused by the rotation of Earth on its axis and the changing length of day and night away from the equator caused by orbiting the sun. Circadian clocks confer advantages by entraining to rhythmic environmental cycles to ensure that internal events within the plant occur at the correct time with respect to the cyclic external environment. Advances in determining the structure of circadian oscillators and the pathways that allow them to respond to light, temperature, and metabolic signals have begun to provide a mechanistic insight to the process of entrainment in Arabidopsis (*Arabidopsis thaliana*). We describe the concepts of entrainment and how it occurs. It is likely that a thorough mechanistic understanding of the genetic and physiological basis of circadian entrainment will provide opportunities for crop improvement.

## Teaser

The mechanisms by which circadian clocks adjust to daily rhythms of light, dark, temperature, and internal metabolism are now coming to light.

## Entrainment is adjustment of circadian oscillators to the rhythms of the environment

Arabidopsis (*Arabidopsis thaliana*), and most likely all plants, have internal circadian rhythms that persist with a period of about 24 hours in constant environments. Circadian rhythms of activity occur because there is in every cell a molecular oscillator with a free-running period of about 24 hours. These molecular circadian oscillators are part of what is usually called a circadian clock, which includes pathways that set the time of the oscillator; the molecular oscillator which generates near 24 hour rhythms; and the output signaling pathways that regulate rhythmic physiology, metabolism, and development. It is thought that plants, and other organisms, evolved these 24 hour circadian clocks as an adaption to the 24 hour rhythms of light/dark and cold/warm caused by the rotation of the Earth on its axis. The biologically relevant phenotypes of circadian systems are the period of the rhythms of activity, which describes the time it takes for one cycle of activity to be completed (∼24 hours), the phase of those activities (when in the cycle an activity occurs), and the amplitude (a measure of the amount of change in activity over time).

Rhythmic environmental stimuli such as light, dark, and temperature can affect the period, phase, and amplitude of the circadian rhythms and are therefore called zeitgebers, from German meaning “time giver.” The major zeitgebers are classically considered to be external light/dark and temperature cycles but have recently been extended to include internal metabolic cycles and other physiological stimuli (Haydon et al., 2013; Webb et al., 2019). There are adaptive advantages for Arabidopsis, and other organisms, in responding to zeitgebers by matching the period and phase of circadian rhythms to the period and phase of the external environmental cycles (Dodd et al., 2005). Circadian entrainment is this matching of internal rhythms to the external cycle of rhythmic zeitgebers. Correct circadian entrainment ensures that internal events occur at the right time with respect to the daily rhythmic environmental cycles and that the plant can anticipate regular daily events like dawn and dusk. In this Update, we describe recent insights about the molecular processes by which zeitgebers entrain the phase and period of circadian rhythms.

## Circadian entrainment can be considered a rapid event or a continuous process

The conceptual understanding of entrainment has mostly been derived from measuring the whole system behavior, that is, the rhythmic activity, rather than discrete molecular events. These concepts have also been influenced by considering biological circadian clocks as analogous to mechanical oscillators, such as pendulums or mechanical clocks (Roenneberg et al., 2003). Considering the system as a mechanical oscillator, we can conceptualize that an entraining zeitgeber might cause an instant adjustment of the circadian oscillator in which there is a sudden change of the entire system from its current phase to a new phase by a discontinuous process. In this discontinuous process, the oscillator instantly adjusts internal time to match the external time of for example, dawn or dusk. In experimental practice, this is measured as a rapid change in the phase of a rhythmic activity in response to a short pulse of an entraining stimuli given in otherwise constant conditions (e.g. a pulse of light provided in otherwise constant dark). Alternatively, circadian oscillators might adapt to entraining stimuli by speeding up or slowing down, resulting in a continuous adjustment to entraining signals. In this view, rather than the oscillator instantly resetting to dawn, the oscillator responds continuously throughout the light period by accelerating and then slowing down in the dark of night (Daan, 2000; Roenneberg et al., 2003). This model of continuous entrainment considers that circadian period constantly changes in response to an entraining stimulus, rather than there being discontinuous discrete changes in phase in response to pulses of a stimulus. In practice, both discontinuous and continuous processes are likely to contribute to entrainment.

Genetic oscillators are not mechanical oscillators and therefore the analogies have limitations in describing the mechanistic basis of entrainment. One aspect that makes biological oscillators different from mechanical oscillators is that the components of the oscillator change over time (e.g. proteins are synthesized and degraded throughout the cycle). For example, in Arabidopsis, the circadian oscillator contains a network of transcriptional regulators whose abundance changes sequentially through the cycle to generate the rhythm. CIRCADIAN CLOCK ASSOCIATED 1/LATE ELONGATED HYPOCOTYL (CCA1/LHY) are two MYB-like transcriptional regulators that peak in abundance near dawn and regulate the expression of the PSEUDORESPONSE REGULATOR (PRR) family of transcriptional repressors that peak in expression through the day and regulate the expression of other oscillator components, including the transcriptional regulator LUX ARRHYTHMO (LUX), which forms a regulatory evening complex (EC) of proteins with EARLY FLOWERING 3 (ELF3) and ELF4 that is active at night. The abundance of these transcriptional regulators is regulated by their transcriptional activities, the activities of other components and through time-of-day specific protein degradation by an E3 ubiquitin ligase complex containing ZEITLUPE (ZTL) (Webb et al., 2019). As an alternative to considering the circadian system as analogous to a mechanical oscillator it is possible to view this network as a signaling pathway in which events occur sequentially (albeit with a high degree of feedback) and there is a bounded plasticity in the timing of these events with respect to each other and the environment (Webb et al., 2019). In this biological view, entrainment might occur through zeitgebers regulating individual components to provide instantaneous, discontinuous entrainment through rapid state changes in components (e.g. protein phosphorylation leading to fast changes in activity) and more continuous entrainment events that speed up or slow down steps in the pathway (e.g. changes in the rate of transcription or translation).

## There are many ways of quantifying circadian entrainment

**Figure 1:**
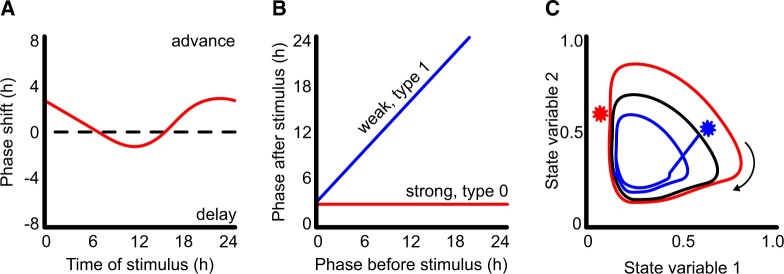
Different approaches allow visualization and analysis of the effects of zeitgebers on circadian oscillators. A, PRCs plot the effect of a stimulus on circadian phase dependent on the time at which a pulse of stimulus is added in otherwise constant conditions. B, Data used to generate PRCs can be plotted as a PTC in which the phase before stimulation is plotted against the phase after stimulation. The angle of the slope indicates the strength of entrainment. C, Limit cycle analysis plots the relationship between variables in the oscillator (e.g. expression levels of oscillator genes) through time in 2D space (black). A stimulus (star) causes phase advance (blue) or delay (red) dependent upon the point in the cycle in which the stimulus is added. In this example, the stimulus that caused the phase advance (blue) initiates the reduction in state variable 1 earlier than would have occurred without perturbation, which is why the phase is advanced. Axes for the limit cycle are normalized arbitrary units.

Different experimental approaches measure the properties of circadian oscillators, the mechanistic basis of entrainment, and the consequence of correct circadian entrainment for the organism (Johnson, 1999). A commonly used approach is to apply pulses of entraining stimuli at different points in the circadian cycle, in otherwise constant conditions, and then measure the degree of resultant phase shift occurring in response to stimulation at each time point in the cycle. For example, a phase response curve (PRC) to light can be created using Arabidopsis plants carrying a circadian promoter:luciferase (LUC) reporter (Covington et al., 2001). The plants are entrained to a light dark cycle and then transferred to constant dark. During the first cycle of constant dark, short pulses of light are applied to batches of plants once an hour and then those plants are returned to constant dark and the subsequent circadian rhythms measured. The timing of an activity for example, peak LUC activity is measured for each plant and the hour difference between the treated and untreated material calculated and plotted against the time of stimulation with light. This generates a PRC (Figure 1A). An alternative approach to reconstruct a PRC is by using a “singularity response” of oscillators that have become desynchronized by prolonged exposure to constant conditions (for Arabidopsis, constant darkness). In this approach, a population of plants is kept for a very long time in constant conditions such that the measured rhythms are very low amplitude and essentially arrhythmic. This apparent arrhythmia and low amplitude are due to the oscillators in a multitude of individual cells becoming desynchronized. A single pulse of entraining stimuli is applied, which resynchronizes the oscillators. Because the resynchronization of multiple desynchronized oscillators involves adjustment from all possible phases, it is possible to mathematically reconstruct the PRC from one treatment at one time point, and therefore this recently described approach, while conceptually complex, has the advantage of reduced experimentation (Masuda et al., 2021).

PRCs describe the magnitude of the change of phase that occurs in response to an entraining signal dependent on the dose and time of stimulation (Figure 1A). An alternative visualization of the same data are phase transition curves (PTCs), in which the phase of an event in the cycle before an entraining stimulus is plotted against the phase after the stimulus (Figure 1B). The slope of the relationship describes the strength of entrainment, with type 1 being a weaker entrainment (near 45° angle to the plot), and type 0 (a near 0° angle) being very strong entrainment.

There are legitimate questions as to whether PRCs and PTCs generated in otherwise constant conditions are representative of the biology in “real world” light and dark cycles. Furthermore, PRCs and PTCs do not account for photoperiod, which also affects entrainment (Roenneberg et al., 2010b), and the magnitude of the phase response in PRCs and PTCs is usually dependent on the strength and duration of stimulation. For these reasons, it can be difficult to predict organismal dynamics in a natural environment from PRCs and PTCs.

To overcome these limitations, entrainment can be investigated in rhythmic environments by measuring the phase angle of an activity in different light and dark cycles (Johnson et al., 2003). The phase angle describes the relative time of an internal event (e.g. peak gene expression) with respect to an external event (e.g. dawn). To ensure an appropriate phase angle between biological activities and the environment, circadian oscillators entrain by adjusting their phase and period to match the timing of rhythmic environmental signals and also respond to seasonal changes (Johnson et al., 2003). However, in rhythmic environments, biological rhythms are usually a composite of circadian regulation and acute responses to light and dark. Thus, circadian regulation can be “masked” by the acute responses to light signals (Bittman, 2021). Masking alone will not generate free-running rhythms in constant conditions because the change in activity is a direct acute response to changes in the environment, and therefore if the environment is constant, the activity will be constant. To study entrainment in rhythmic environments, like those found in nature, it is necessary to separate circadian effects from those caused by masking. Happily, a basic property of circadian clocks allows entrainment to be observed and separated from the effects of masking. In circadian systems, a shift in phase will occur if the endogenous circadian period (*τ*) differs to the period of the environmental rhythm (*T*). The magnitude of that phase shift will be equal to the difference in period of the circadian oscillator to that of the environmental cycle (*τ* − *T*). This means that when stable entrainment is achieved (*τ* = *T*), the phase angle will be stable. In practice, this means that if the circadian period differs from that of the environment the phase angle will be unstable, demonstrated by peaks of activity occurring at different times in each successive cycle (Johnson et al., 2003). Such experiments allow the range of entrainment to be investigated. For example, the phase angle of *CHLOROPHYLL A/B BINDING 2 promoter:LUC* (*CAB2::LUC*) reporter activity was stable in a long period mutant of *ZEITLUPE* (*ztl-1*; *τ* = 28) in a 14 hours light, 14 hours dark-light cycle (T28), suggesting *ztl-1* plants can entrain to T28 but was unstable in T20 (10 hours light, 10 hours dark), suggesting that *ztl-1* cannot entrain to a 20 hour environmental cycle (Dodd et al., 2014), possibly explaining why *ztl-1* grows better in T28 than T20 (Dodd et al., 2005).

Time shift experiments in which the day or night are unexpectedly artificially extended can indicate the importance of circadian entrainment in regulating outputs of the oscillator and separates circadian regulation from potential masking effects of light signaling. Such an approach was used to demonstrate the importance of circadian timing in the regulation of the degradation of diel starch reserves in Arabidopsis leaves (Graf et al., 2010).

## Formalisms allow the complex processes of entrainment to be simplified

The complex changes in behavior that occur in a circadian oscillator during entrainment can be simplified as limit cycle models in which the network of oscillating components changing over time are reduced to measurements of state variables that move through a 2D phase plane in a stable cycle around a stationary point or singularity (Figure 1C;Johnson et al., 2003). Since a state variable describes any quantity that is sufficient to calculate the future behavior of a dynamical system if left unperturbed, many different components of a mathematical model of a circadian oscillator could be plotted in a limit cycle. An intuitive example is to plot the relative abundance of transcripts encoding oscillator components over time. For example, we have plotted the abundance of *PRR7/9* against *CCA1/LHY*, the reciprocal regulation between the proteins encoded by these sets of transcripts means that when *CCA1/LHY* abundance is high, *PRR7/9* tend to be low and as *CCA1/LHY* decrease through the day, the levels of *PRR7/9* increase, describing a cyclic relationship in which one cycle is equal to 24 hours (Ohara et al., 2018). In constant conditions, the limit cycles continue on the same trajectory, such that each cycle plot overlies the previous cycle. Perturbation by zeitgebers affects the trajectory of the limit cycle (Figure 1C) such that when the state variable returns to the limit cycle, it does so at a different phase to that without perturbation. Importantly, the trajectory of limit cycles can be different in altered environments, accounting for different entrainment responses in varied conditions, such as constant light or constant dark (Roenneberg et al., 2010a).

## Light is a primary zeitgeber that causes transcriptional changes in the Arabidopsis circadian oscillator near dawn

**Figure 2:**
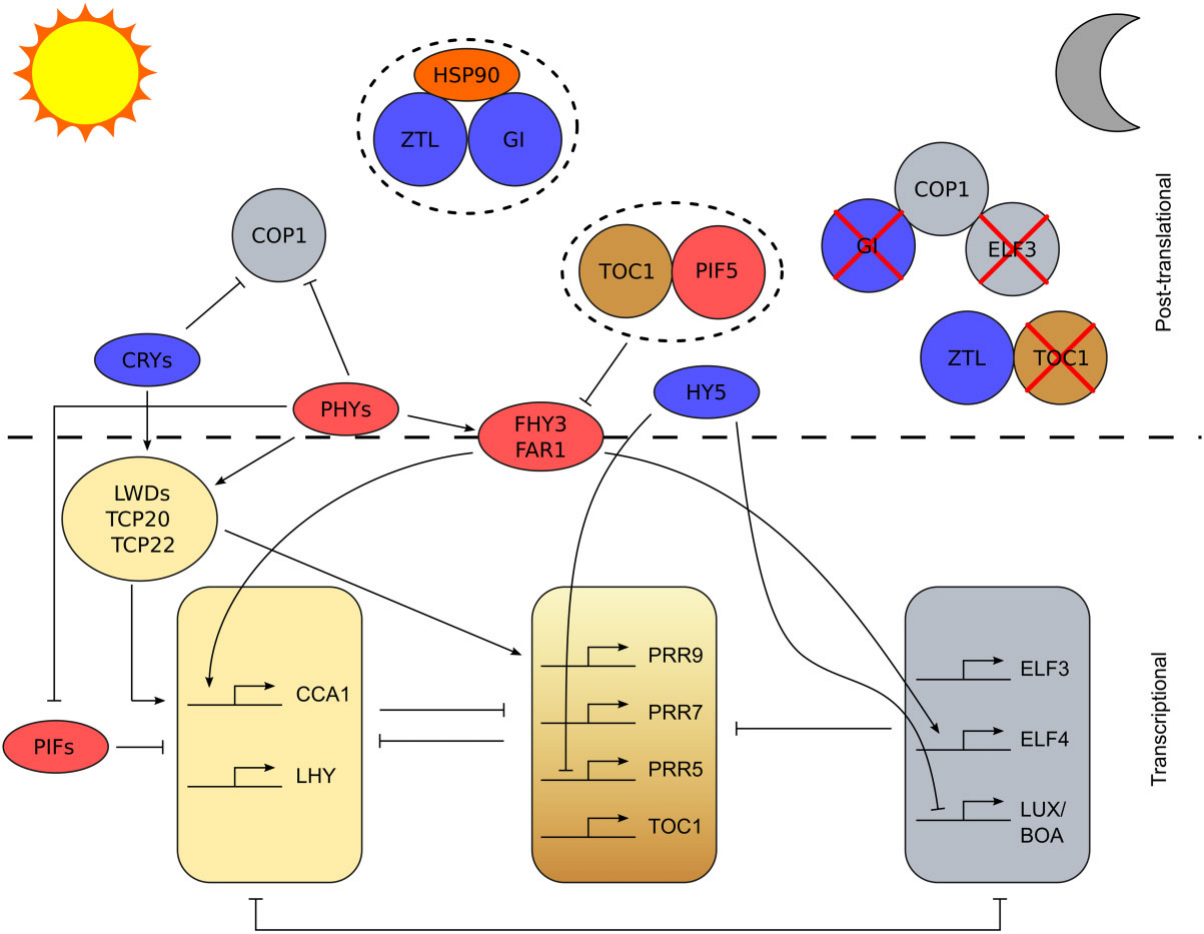
Mechanisms of light entrainment of the Arabidopsis circadian oscillator. Simplified schematic diagram illustrating different mechanisms by which light might entrain the Arabidopsis circadian oscillator. The transcriptional oscillator is depicted in the bottom half of the figure with components grouped as previously described (Fogelmark and Troein, 2014). Pointed arrow heads represent activation, flat heads represent repression. The top half of the figure outlines examples of posttranslational regulation with components in circles being involved in this type of regulation. Blue background indicates the protein is involved in blue light regulation of the oscillator and red indicates association with red light signalling of the clock. Dashed oval surrounding components paired together indicates stabilization, red cross indicates that component is degraded by the direct or indirect action of its uncrossed neighbor. The figure is approximately organized by time of day going from dawn on the left (sun) to night on the right (crescent moon).

Entrainment to dawn is primarily achieved through transcriptional regulation of circadian oscillator transcript abundance (Flis et al., 2016). In the Arabidopsis circadian oscillator, there are multiple points where light information can be integrated into a change in circadian gene transcript abundance (Figure 2). Red light is detected by five phytochromes (PHYA–E), while blue light is detected by the cryptochromes (CRY1 & 2) and ZTL. Loss-of-function mutations of *PhyA* and *PhyB* increase circadian period in constant light because reduced light input due to loss-of-function mutations of photoreceptors, or low light levels, increases free-running circadian period (Somers et al., 1998). The phytochromes have multiple roles in the system binding to both oscillator proteins and indirectly to gene promoters but the mechanisms of transduction of the red-light signal to oscillator regulation are not fully resolved (Sanchez et al., 2020). PHYTOCHROME INTERACTING FACTORs (PIFs) form important signaling hubs in Arabidopsis which act downstream from the PHYs (Sanchez et al., 2020). The PIFs associate with the *CCA1/LHY* promoters (Martínez-García et al., 2000; Oh et al., 2012) and bind PRR5,7,9 proteins (Martín et al., 2018), GIGANTEA (GI) (Nohales et al., 2019), and TIMING OF CAB EXPRESSION 1 (TOC1) (Soy et al., 2016; Zhu et al., 2016; Liu et al., 2020) *in vivo*. Exposure of Arabidopsis seedlings to pulses of white light at different times prior to dawn causes greater phase delays in *CCA1* promoter activity in *pif4-101 pif1-1* mutants compared with wild-type indicating a role for PIFs in light input to the clock, possibly through their interaction with the *CCA1* and *LHY* promoters (Nohales et al., 2019). Light-mediated induction of *CCA1/LHY* expression affects the phase and period of the oscillator by regulating other components, as shown by artificial ethanol-induced expression of *CCA1* at dusk, which causes a 9 hour phase shift in *CAB2::LUC* activity that persists for up to 13 days in constant light (Knowles et al., 2008). FAR-RED ELONGATED HYPOCOTYL 3 (FHY3) is another signaling intermediate acting downstream of the phytochromes (Siddiqui et al., 2016) involved in *CCA1* induction and plays a crucial role in gating the response to light such that the circadian oscillator is most sensitive to light at dawn (Allen et al., 2006; Liu et al., 2020). Modulation of the effectiveness of inputs to the oscillator by gating from the oscillator is feedback that prevents continuous resetting of the oscillator by ensuring there is a “dead zone” in which the oscillator is unresponsive to a zeitgeber at certain times of day (Figure 1A). Full light activation of *CCA1* expression requires TEOSINTE BRANCHED 1, CYCLOIDEA, PCF (TCP)-DOMAIN FAMILY PROTEIN 20 (TCP20), TCP22, and LIGHT-REGULATED WD 1 (LWD1) (Wu et al., 2016). The transcriptional regulators TCP20 and TCP22 bind to the TCP binding site (TBS) motif in the *CCA1* promoter and in the presence of LWD1 or LWD2 cause an increase in *CCA1* expression. Loss-of-function mutations of *TCP20*, *TCP22*, or the *LWDs* reduce free-running circadian period and cause a phase advance of *CCA1::LUC* activity in diel conditions (Wu et al., 2016), indicative of their role in entrainment.

Reduced blue light input in *cry1 cry2* double mutants also increases free-running circadian period (Devlin and Kay, 2000). The mechanisms by which the cryptochromes transduce blue light information to the circadian oscillator are also poorly understood and might also involve PIFs (Sanchez et al., 2020). Recently it has been reported that CRY2 associates with the mRNA ADENOSINE METHYLASE (MTA) subunit of METTL3/14-type *N*^6^-methyladenosine RNA methyltransferase (m^6^A writer) to facilitate m^6^A RNA methylation (Wang et al., 2021). In Arabidopsis, m^6^A methylation typically increases the stability of transcripts (Anderson et al., 2018). Loss-of-function mutations of *mta* or *cry1 cry2* cause a decrease in *CCA1* transcript half-life and an increase in free-running circadian period (Wang et al., 2021). Blue light stimulates liquid–liquid phase separation of CRY2, forming CRY2 photobodies and facilitating binding to MTA, suggesting that CRY dependent m^6^A RNA methylation provides a mechanism for blue light entrainment (Wang et al., 2021). However, *mta* mutants have much less effect on free-running circadian period than *cry1 cry2* mutants, suggesting additional mechanisms of CRY action are required for entrainment. Photobody formation might also contribute to red light signaling because mutation of *PHOTOPERIODIC CONTROL OF HYPOCOTYL 1*, which is required for PhyB photobody formation, disrupts the otherwise robust oscillations of *CCA1::LUC* in continuous darkness in a constitutively active *phyB* mutant (Huang et al., 2019). Blue light also probably influences expression of circadian clock genes through transcriptional and posttranslational activation of *ELONGATED HYPOCOTYL 5* (*HY5*) and its homolog *HY5-HOMOLOG* (*HYH*) (Hajdu et al., 2018). These two basic leucine zipper domain (bZIP) transcription factors bind to the promoter regions of *CCA1*, *PRR9*, *PRR5*, *LUX*, *ELF3*, *ELF4*, and *TOC1* and expression of *PRR5* and *LUX* are upregulated in the short-period *hy5 hyh* double mutant (Hajdu et al., 2018).

Light signaling also alters chromatin state. Upon activation, the far-red sensing PhyA binds chromatin at target circadian *loci* (Chen et al., 2014). The circadian oscillator protein GI is a positive regulator of light signaling that decreases the stability of PIF3 and likely affects its ability to bind to chromatin (Nohales et al., 2019). In *gi-2* mutants, the amplitude of *CCA1* expression is decreased and free-running circadian period is shortened compared with wild-type plants but phenotypes are rescued in the presence of loss-of-function mutations of *PIF3*, *PIF4*, and *PIF5*, demonstrating that the PIFs repress *CCA1* in a way that alters circadian oscillator function (Nohales et al., 2019). Light-regulated transcriptional splicing variation might also affect entrainment. A 2 hour pulse of white light provided during the middle of the night to simulate a long day photoperiod, altered the splicing of 382 transcripts including *LHY*, *REVEILLE 8*, *TIME FOR COFFEE*, *JUMONJI DOMAIN CONTAINING 5*, and *CASEIN KINASE II BETA CHAIN 3* (Mancini et al., 2016). While it is broadly agreed that CRYs and PHYs participate in light entrainment, an Arabidopsis *phyA phyB cry1 cry2* quadruple mutant is able to entrain to an antiphase photoperiod despite being developmentally insensitive to light, demonstrating that light signaling for development and entrainment is separable and that circadian entrainment by light is possible by alternative pathways (Yanovsky et al., 2000).

## Posttranslational events contribute to entrainment of the Arabidopsis circadian oscillator to dusk

It is necessary for there to be light inputs to the circadian oscillator at both dawn and dusk to allow measurement of photoperiod, suggesting that both dawn and dusk are entrainment cues (Locke et al., 2005). Increased photoperiod results in later phase of peak oscillator transcript abundance with respect to dawn, demonstrating that dusk is an entrainment signal (Flis et al., 2016; Hearn et al., 2018). “Dusk sensitivity” (Edwards et al., 2010) is an important aspect of entrainment in Arabidopsis, with some components, such as *CCA1* and *LHY* fixed to dawn and others such as *TOC1* fixed to dusk. Other components respond to the timing of both dawn and dusk, conferring greater plasticity of phase (Dixon et al., 2014).

In contrast to light-mediated transcriptional regulation at dawn, entrainment at dusk might be primarily dependent upon posttranslational regulation of proteins and their relative stability in light and dark (Flis et al., 2016). In the dark following dusk, GI dissociates from ZTL, which allows ZTL to target TOC1, PRR5, and CCA1 HIKING EXPEDITION for proteasomal mediated degradation (Pudasaini et al., 2017). ZTL is a light, oxygen, or voltage (LOV) domain blue light photoreceptor. In the presence of blue light, GI binds to the LOV domain of ZTL and recruits HEAT SHOCK PROTEIN 90 (HSP90), which facilitates maturation of ZTL and counteracts its degradative activity (Cha et al., 2017; Lee et al., 2019). Thus, at dusk, there is a change in state of the circadian oscillator caused by ZTL mediated degradation of at least three components (Figure 2).

**Figure 3:**
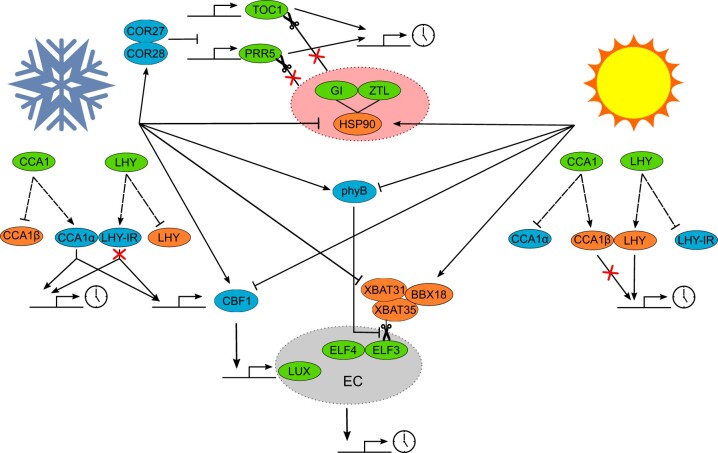
Simplified overview of potential temperature entrainment mechanisms. Schematic showing how cold (snowflake) and heat (sun) are transduced to the circadian clock. Circadian clock proteins are denoted by green ovals, components upregulated in the cold denoted by blue ovals, and components upregulated in the heat denoted by orange ovals. Dashed lines from CCA1 and LHY denote alternative splicing with CCA1α retaining the ability to bind to target DNA sequences and LHY-1R representing intron retaining splice variants that are targeted for nonsense-mediated decay (NMD). Pointed arrow heads represent activation, blunt arrow heads represent repression, and scissors represent proteasomal mediated degradation of proteins. Large red oval containing GI-ZTL-HSP90 represents complex where ZTL is unable to ubiquitinate PRR5 and TOC1. Gray oval labeled EC represents the EC where ELF3 is the target of degradation by the XBAT31–XBAT35–BBX18 complex but is stabilized by PhyB.

Another event that occurs near dusk is the formation of an EC of ELF3, ELF4, and LUX proteins (Figure 3). The EC is an integrator of environmental information to the oscillator, including light and temperature (Huang and Nusinow, 2016). Expression of *ELF3* and *ELF4* is induced by the PhyA signaling intermediates FAR-RED IMPAIRED RESPONSE 1 (FAR1), FHY3 and HY5 directly binding to the *ELF4* promoter in the light (Li et al., 2011). Also in the light, PhyB associates with and stabilizes ELF3 protein (Liu et al., 2001). Following dusk, in the dark, CONSTITUTIVE PHOTOMORPHOGENESIS 1 (COP1), an E3 ubiquitin ligase, binds and ubiquitinates ELF3, targeting it for degradation (Yu et al., 2008). Prior to the degradation of ELF3, the COP1–ELF3 complex binds to GI in the dark, allowing COP1 to ubiquitinate GI for proteasomal degradation (Yu et al., 2008). Loss-of-function *elf3* mutants are unable to entrain to light and dark cycles of different periods and have increased magnitude of response to pulses of light at different points in the circadian cycle, suggesting entrainment defects (Kolmos et al., 2011). Furthermore, loss-of-function mutations of *ELF3*, *ELF4*, or *LUX* lead to complete circadian arrhythmia in continuous conditions (Nagel and Kay, 2012).

## Temperature cycles entrain the Arabidopsis circadian oscillator through a mechanism that might include regulation of transcript splicing

A characteristic feature of circadian clocks is temperature compensation, which refers to their ability to maintain a similar free-running circadian period across a physiological range of temperatures (Gould et al., 2006). It is somewhat counterintuitive that while the Arabidopsis circadian oscillator buffers free-running period against temperature changes, it can also entrain to rhythmic temperature cycles (Figure 3). A difference of just 4°C between day and night is sufficient for temperature entrainment (Thines and Harmon, 2010). Three-hour pulses of 27°C in plants entrained at 22°C can advance phase by 7 hours in the morning and delay phase to a similar extent around dusk, demonstrating the potential for temperature to be a zeitgeber (Davis et al., 2018). In contrast to light signaling, where photoreceptors have been well characterized, plant thermosensors are still being identified. Ambient temperature is typically higher during the photoperiod, which might explain why light and temperature entrainment share common components, such as PhyB acting as a potential thermosensor (Vu et al., 2019). Humidity is closely linked to temperature with relative humidity typically increasing in the evening as temperature decreases. Oscillations in relative humidity can entrain the circadian oscillator but result in an antiphase entrainment, such that *CCA1::LUC* activity is greatest at subjective dusk and *TOC1::LUC* activity is greatest at subjective dawn (Mwimba et al., 2018). This suggests in diel conditions relative humidity is not a critical zeitgeber.

Temperature also regulates the oscillator through changes in the abundance of transcripts encoding oscillator components. A decrease in temperature triggers the expression of cold-responsive genes which in turn alter the expression of oscillator genes. The cold-induced C-REPEAT BINDING FACTOR 1 (CBF1), which is positively regulated by CCA1 and LHY (Dong et al., 2011), induces expression of *LUX* (Chow et al., 2014)*.* CBFs also induce expression of *COLD RESPONSIVE 27* (*COR27*) and *COR28*, which associate with the chromatin regions of *PRR5* and *TOC1* to repress their expression (Li et al., 2016). Loss-of-function mutants of *COR27* and *COR28* have increased free-running circadian period. COR27 and COR28 are also regulated by blue light, which represses their expression but stabilizes the proteins (Li et al., 2016), which are otherwise degraded by COP1 in the dark (Li et al., 2020).

Temperature-dependent splicing and nonsense-mediated decay (NMD) might transduce temperature signals to alter the phase of the oscillator (Staiger and Green, 2011; James et al., 2012; Mateos et al., 2018). Loss-of-function mutations of the splicing apparatus can alter free-running circadian period. Mutation of *PROTEIN ARGININE METHYL TRANSFERASE 5*, which demethylates histone 4 and various splicing factors including SMALL NUCLEAR RIBONUCLEOPROTEIN D1 (SmD1), SmD2, and SM-LIKE PROTEIN 4 (Deng et al., 2010), causes an increase in free-running circadian period due to increased retention of intron 3 in *PRR7* and consequently decreased abundance of full-length protein (Hong et al., 2010; Sanchez et al., 2010). Similarly, mutations of *SNW/SKI INTERACTING* (*SKIP*) (Wang et al., 2012) and *SPLICEOSOMAL TIMEKEEPER LOCUS 1* (*STIPL1*) (Jones et al., 2012) increase free-running circadian period. Mutation of *SKIP* increases the abundance of aberrant splice variants of *PRR7* and *PRR9*, although the increased free-running period phenotype diminishes with increasing temperature (Wang et al., 2012). Mutation of *STIPL1* alters the alternative splicing of *CCA1*, *LHY*, *PRR9*, *GI*, and *TOC1* (Jones et al., 2012). Transcripts of *PRR7*, *PRR9*, *ELF3*, and *TOC1* undergo temperature-dependent alternative splicing reliant on gem nuclear organelle associated protein 2 (GEMIN2), which facilitates formation of small nuclear ribonucleoprotein particles (snRNPs) involved in splicing (James et al., 2012). Loss of functional *GEMIN2* causes increased intron retention within *TOC1*, *PRR7*, *CCA1*, and *LHY*, a decreased free-running circadian period and decreased temperature compensation (Schlaen et al., 2015).

A decrease in temperature to 4°C causes a two-fold increase in *CCA1α* transcript abundance and an 80% decrease in *CCA1β* transcript abundance (Seo et al., 2012). As *CCA1β* is missing the MYB-domain which is required for DNA binding, but retains the ability to dimerize with *CCA1α* and *LHY*, the *CCA1α:CCA1β* ratio has a direct impact on the functioning of the oscillator; increased *CCA1β* decreases free-running circadian period (Seo et al., 2012). Similarly, total *LHY* transcript abundance decreases at cool temperatures due to temperature-dependent alternative splicing increasing transcripts containing the 5′-untranslated region and exon 5a which are targets for NMD (James et al., 2012). Changes in *LHY* transcript are detected after just 1 hour of cold treatment and in response to a 1°C decrease in temperature (James et al., 2018) dependent on several splicing factors including *POLYPYRIMIDINE TRACT-BINDING PROTEIN 1* (*PTB1*), *PTB2*, and *U2 SMALL NUCLEAR RIBONUCLEOPROTEIN AUXILIARY FACTOR 65* (*U2AF65A*) (James et al., 2018). *SICKLE* is another important component of the temperature-dependent splicing response with loss-of-function mutations leading to accumulation of *LHY* and *CCA1* splice variants along with a decrease in rhythmicity and increased period under continuous conditions (Marshall et al., 2016).

Whether the changes in transcription and transcript splicing contribute to entrainment to temperature cycles is not yet resolved. However, the repressive EC formed of ELF3, ELF4, and LUX proteins in Arabidopsis is required for temperature entrainment (Thines and Harmon, 2010). In the absence of a functional EC, expression of *PRR7*, *PRR9*, *GI*, and *LUX* is high, irrespective of temperature. Whereas in the wild-type, the abundance of these transcripts increases in response to high temperatures. This suggests that a role of the EC is to repress *PRR7*, *PRR9*, *GI*, and *LUX* at lower temperatures (Mizuno et al., 2014). Of the three proteins in the EC, only LUX directly binds DNA, and this is temperature dependent (Silva et al., 2020). Thermoresponsiveness of the EC is conferred by ELF3 and ELF4. ELF4 stabilizes the LUX–ELF3 interaction, which becomes increasingly important at higher temperatures (Silva et al., 2020). ELF3 contains a polyglutamine repeat of varying lengths, embedded within a prion-like domain, which allows ELF3 to switch between an active soluble form at lower temperatures and an inactive multimeric form at higher temperatures (Jung et al., 2020). The stability of ELF3 is also modulated by the activity of the E3-ubiquitin ligases XB3 ORTHOLOG 1 IN ARABIDOPSIS THALIANA (XBAT31) (Zhang et al., 2021b) and XB3 ORTHOLOG 5 IN ARABIDOPSIS THALIANA (Zhang et al., 2021a) which promote the ubiquitination and subsequent 26S proteasomal degradation of ELF3 at warm temperatures in a B-BOX 18 (BBX18) dependent manner.

## Light and temperature signaling pathways converge to entrain circadian oscillators

The EC is required for both correct light and temperature entrainment, demonstrating convergence of these signaling pathways at components of the circadian oscillator. Similarly, there is convergence at PhyB. An increase in temperature leads to the thermal reversion of PhyB from the active PFr form to the inactive Pr form (Legris et al., 2016). Changes in the PFr:Pr ratio coupled with inactivation of the EC at high temperatures lead to increased expression of *PIF4*, which is a key hub in thermomorphogenesis (Box et al., 2015; Raschke et al., 2015).

Mutation of HSP90, a co-chaperone of ZTL, renders the circadian oscillator less sensitive to 3 hour pulses of warmth around dawn, suggesting that functional HSP90 is required for morning entrainment to temperature cycles (Davis et al., 2018). In addition to its role in regulating the degradation of TOC1 and PRR5, the ZTL-GI-HSP90 complex is also important for maintaining protein integrity by facilitating the renaturing of proteins or polyubiquitylating them for proteasomal mediated degradation at high temperatures (Gil et al., 2017). Mutation of *ZTL* is sufficient to reduce thermotolerance in Arabidopsis (Gil et al., 2017). A number of other signals converge on the ZTL-GI-HSP90 complex to regulate its activity and thus influence the circadian oscillator, including tetrapyrroles produced in plastids, which inhibit the activity of HSP90 and consequently decrease the stability of ZTL (Norén et al., 2016).

## Internal metabolic signals can entrain the Arabidopsis circadian oscillator

Internal stimuli such as the hormones abscisic acid (Lee et al., 2016) and ethylene (Haydon et al., 2017), cations such as Ca^2+^ (Martí Ruiz et al., 2018), Fe^3+^ (Salomé et al., 2013), and Mg^2+^ (Webb et al., 2019; de Melo et al., 2021), and metabolites such as sucrose and glucose (Haydon et al., 2013) can affect the free-running circadian period and phase of the Arabidopsis circadian oscillator. Of these, only sucrose has been shown formally to be a potential entraining stimulus (Haydon et al., 2013).

**Figure 4:**
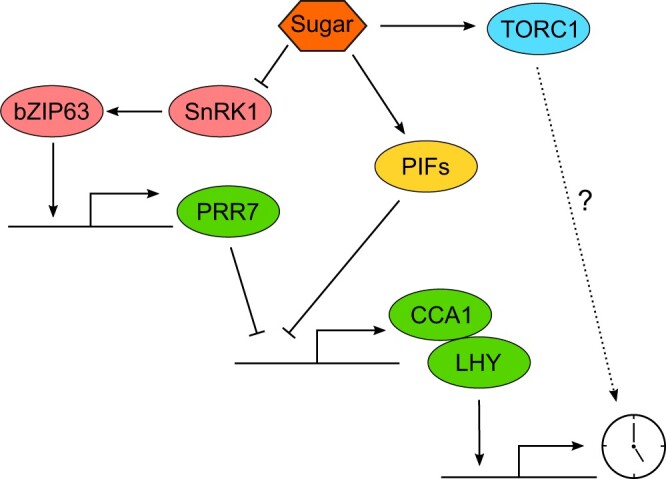
Entrainment of the Arabidopsis circadian oscillator by sugar schematic indicating how sugar can alter the expression of the core clock components *CCA1* and *LHY*. Arrowheads represent activation, blunt heads represent repression, and dashed line represents possible interaction.

Sucrose is the major product of photosynthesis, providing a source of fixed carbon and energy for growth and respiration. Under energy-depleted conditions, such as in low light intensity, sucrose pulses can entrain the circadian clock, inducing phase advances near dawn. The addition of a continuous supply of sucrose to plants in energy-depleted conditions also reduces the free-running circadian period but there is no period reduction in response to sucrose under energy replete conditions (Haydon et al., 2013). When sugar levels are low, the repression of SUCROSE NON-FERMENTING1 RELATED PROTEIN KINASE 1 (SNRK1) by the signaling sugar trehalose-6-phopshate is released, allowing the SNF1 KINASE HOMOLOG 10 subunit of SNRK1 to phosphorylate bZIP63, which in turn binds to the ACGT-core binding motifs within the *PRR7* promoter to induce *PRR7* expression (Frank et al., 2018). Mutation of any component of this signaling pathway reduces sensitivity to morning pulses of sucrose (Frank et al., 2018). The responsiveness of the circadian oscillator to sucrose is also altered in the *pif1 pif3 pif4 pif5* quadruple mutant (*pifQ*), where addition of increasing concentrations of sucrose reduces free-running circadian period less in *pifQ* plants compared with wild type (Shor et al., 2017). Binding of the PIFs to the *LHY* promoter and PIF5 to the *CCA1* promoter is enriched in the presence of sucrose but the PIFs do not bind to the *PRR7* promoter, raising the possibility that the PIFs might provide a *PRR7*-independent mechanism of sucrose signaling to *CCA1/LHY* (Figure 4).

*TARGET OF RAPAMYCIN* (*TOR*) might also contribute to sensing of metabolic energy status by the circadian oscillator. TOR is a kinase that associates with the REGULATORY-ASSOCIATED PROTEIN OF TOR and LETHAL WITH SEC THIRTEEN 8 (LST8) to form the TOR COMPLEX 1 (TORC1) that has a central role in adapting plant growth to environmental inputs and the local energy status (Caldana et al., 2019). Inhibition of TOR lengthens free-running circadian period in continuous low-intensity red light (Wang et al., 2020) and inhibits the response of the circadian oscillator to sucrose (Zhang et al., 2019). These results, coupled with the observation that TOR regulation of root cell proliferation is dependent upon functional *PRR5*, *PRR7*, and *PRR9* (Li et al., 2019), indicates that under energy replete conditions the TORC1 might be important for signaling to the circadian oscillator.

In complete darkness, which depletes internal sugar stores, oscillations of the circadian oscillator are not robust and dampen quickly, but can be restored by the addition of exogenous sucrose (Dalchau et al., 2011). The restoration of circadian rhythms in constant darkness is due to the stabilization of GI protein (Haydon et al., 2017). When sucrose is reapplied to plants after a prolonged period of darkness, the phase of the re-established oscillator is dependent on the time of day at which the sugars were first supplied, providing further evidence that sugars are a zeitgeber.

It is not clear why the Arabidopsis circadian oscillator is responsive to sugars. We proposed that sugars from photosynthesis might set the circadian oscillator to a “metabolic dawn,” one of multiple resetting events that occur through the circadian cycle in addition to the environmental dawn and dusk (Haydon et al., 2013). However, at least in the square wave light of growth cabinets, light signaling dominates over sugar signals to set circadian phase (Moraes et al., 2019), and mathematical models that do not explicitly include sugar signaling can describe entrainment to different photoperiods (Flis et al., 2016), which might indicate sugar signaling is not important for setting circadian phase. On the other hand, mutants that affect the response of the circadian oscillator to sugars, including *prr7-11* and *bZIP63-1*, can have a late phase in light-dark cycles, indicative of a role for sugars in setting circadian timing (Frank et al., 2018). Additionally, *prr7-11* mutants affect the diel turnover of transient starch reserves (Seki et al., 2017), which might indicate a role for sugar sensing by the circadian oscillator in timing this important process.

*PRR7* seems to be important in regulating circadian period in response to other signals in addition to sucrose. Decreasing red light intensity or addition of nicotinamide increase circadian period in wild-type plants, but these responses are absent in *prr7* loss-of-function mutants (Farré et al., 2005; Mombaerts et al., 2019). Thus, while an effect of *PRR7* on diel starch turnover might be indicative of a role for sugar-entrainment of the circadian oscillator participating in regulation of the diel turnover of transient starch reserves (Seki et al., 2017), it is possible that the effects of *PRR7* on diel starch turnover could be related to responses of *PRR7* to entraining stimuli other than photosynthetic sugars and models that do not incorporate a role for sugar signaling to the circadian oscillator in the regulation of the diel turnover of starch are also credible (Scialdone et al., 2013). Further work is required to determine why the pathways involving SNRK1 and bZIP63 that respond to low energy and that which involves TOR, which responds to energy replete conditions, can affect circadian timing and entrainment (Figure 4).

## The mechanistic basis of circadian entrainment is not fully described

Understanding the mechanisms by which circadian oscillators entrain to the environment is as physiologically relevant as understanding oscillator structure and how rhythms are generated. Mathematical models suggest that in the morning entrainment to light occurs mostly through transcriptional regulation, whereas at dusk posttranslational regulation is most important in setting the circadian time (Figure 2; Flis et al., 2016). The relative roles of red and blue light input to the oscillator have not been formally incorporated into a mechanistic mathematical model of circadian en)trainment, partly because the pathways are very complex and also because there is crosstalk between red and blue light signaling to the oscillator (Balcerowicz et al., 2021). The mechanistic basis of temperature entrainment of the oscillator is poorly understood and also not incorporated into mathematical models. Because temperature affects nearly all biological processes, determining those events that specifically contribute to entrainment is challenging (Figure 3). The pathways by which metabolites regulate the oscillator are becoming clearer (Figure 4), with bZIP63 transcriptional regulation of *PRR7* signaling low sugar and energy status and TOR contributing to inputs from replete sugar signaling. However, the extent and purpose of entrainment to sugar signals is a matter of debate (Moraes et al., 2019; Webb et al., 2019). *PRR7* seems to be important in integrating light and metabolic signals to the oscillator but the mechanism by which *PRR7* has such profound effects, distinct from the other *PRR*s, is not well understood (see “Outstanding Questions”).

There is a need for detailed molecular studies that describe specific molecular events that occur in the oscillator during entrainment to rhythmic environments. Most studies have focused on transcriptional changes, but additional investigation is needed to identify the rapid molecular events, such as protein phosphorylation, that might contribute to rapid changes of phase that are thought to occur during discontinuous entertainment. These rapid molecular events could be identified by measuring molecular responses to pulses of entraining stimuli in otherwise constant conditions. Pharmacological or genetic interventions that inhibit molecular changes in response to rapid stimulation should reduce or prevent a change in circadian phase if the identified events are bona fide contributors to circadian entrainment. Similarly, experimental and mathematical analysis of the plasticity of circadian period in response to changes in light intensity, or metabolites such as sugars, will provide insight into the molecular basis of continuous entrainment. *PRR7* is a candidate for a central regulator of continuous entrainment because *prr7* mutants reduce the ability of the oscillator to change free-running circadian period in response to light, sugars and nicotinamide (Farré et al., 2005; Haydon et al., 2013; Mombaerts et al., 2019). How *PRR7* has such profound effects on changes in free-running circadian period in response to changes in light, sugar, or nicotinamide treatment strength is not known. The goal of these investigations will be to achieve a quantitative description, such that it will be possible to predict the degree of change in circadian phase and period based on measured changes in abundance or activity of components of the oscillator, and vice versa, predict the change in molecular abundance and activity of specific oscillator components based on the measured change in circadian phase of output pathways.

The crosstalk and feedback in the signaling pathways activated by multiple endogenous and external cues make unraveling the entrainment system difficult and it is particularly hard to interpret the laboratory data concerning entrainment in a field context, especially because shading from neighbors in dense monocultures affects circadian phase, at least in field-grown sugarcane (*Saccharum* hybrid) (Dantas et al., 2021). A challenge for the next decade is to be able to use the mechanistic insight made in the laboratory to predict the performance of plants in field conditions and use this knowledge to improve crop yields through the practice of chronoculture (Steed et al., 2021).

Advances

- Transcriptional regulators that are part of the rhythm-generating circadian oscillator have been identified and are well characterized.
- Mathematical models based on prior knowledge of the components and the timing of interactions fit training data well and have good predictive power for circadian dynamics.
- At dawn there is a wave of transcriptional regulation by red and blue light signaling pathways.
- At dusk, posttranslational regulation affects the stability of circadian oscillator proteins.
- Considering entrainment as transcriptional regulation at dawn and posttranslational regulation at dusk is sufficient to describe entrainment of the Arabidopsis circadian oscillator to changes in photoperiod.

Outstanding questions

- What are the molecular signaling pathways by which red and blue light regulate the transcription of circadian oscillator genes?
- Temperature affects many processes associated with the circadian oscillator, but how does this bring about entrainment?
- What is the physiological role for interactions between temperature and light signaling pathways in regulation of the circadian oscillator?
- Why does the circadian oscillator respond to sugar signals?
- What are the key molecular changes in the oscillator that adjust circadian phase?
- Can quantifying the effect of specific molecular events on the phase of the oscillator provide insight into the basic processes of circadian entrainment?

## Funding

S.W. is supported by the China Scholarship Council award 201908420338. G.S. is supported by the UK Reasearch and Innovation Biotechnology and Biological Sciences Research Council Grant BB/S006370/1 awarded to A.A.R.W.

## Data availability statement

New data are not associated with this manuscript.

*Conflict of interest statement*. None declared.

## Notes

All authors contributed equally, wrote the text, and prepared the figures.

The author responsible for distribution of materials integral to the findings presented in this article in accordance with the policy described in the Instructions for Authors (https://academic.oup.com/plphys/pages/general-instructions) is: Alex A.R. Webb (aarw2@cam.ac.uk).
